# The Effect of Dopaminergic Medication on Beat-Based Auditory Timing in Parkinson’s Disease

**DOI:** 10.3389/fneur.2016.00019

**Published:** 2016-02-22

**Authors:** Daniel J. Cameron, Kristen A. Pickett, Gammon M. Earhart, Jessica A. Grahn

**Affiliations:** ^1^Brain and Mind Institute, University of Western Ontario, London, ON, Canada; ^2^Occupational Therapy Program, Department of Kinesiology, University of Wisconsin-Madison, Madison, WI, USA; ^3^Program in Physical Therapy, Department of Neuroscience, Department of Neurology, Washington University School of Medicine in St. Louis, St. Louis, MO, USA; ^4^Department of Psychology, University of Western Ontario, London, ON, Canada

**Keywords:** beat perception, rhythm, timing, Parkinson’s disease, dopamine

## Abstract

Parkinson’s disease (PD) adversely affects timing abilities. Beat-based timing is a mechanism that times events relative to a regular interval, such as the “beat” in musical rhythm, and is impaired in PD. It is unknown if dopaminergic medication influences beat-based timing in PD. Here, we tested beat-based timing over two sessions in participants with PD (OFF then ON dopaminergic medication) and in unmedicated control participants. People with PD and control participants completed two tasks. The first was a discrimination task in which participants compared two rhythms and determined whether they were the same or different. Rhythms either had a beat structure (metric simple rhythms) or did not (metric complex rhythms), as in previous studies. Discrimination accuracy was analyzed to test for the effects of beat structure, as well as differences between participants with PD and controls, and effects of medication (PD group only). The second task was the Beat Alignment Test (BAT), in which participants listened to music with regular tones superimposed, and responded as to whether the tones were “ON” or “OFF” the beat of the music. Accuracy was analyzed to test for differences between participants with PD and controls, and for an effect of medication in patients. Both patients and controls discriminated metric simple rhythms better than metric complex rhythms. Controls also improved at the discrimination task in the second vs. first session, whereas people with PD did not. For participants with PD, the difference in performance between metric simple and metric complex rhythms was greater (sensitivity to changes in simple rhythms increased and sensitivity to changes in complex rhythms decreased) when ON vs. OFF medication. Performance also worsened with disease severity. For the BAT, no group differences or effects of medication were found. Overall, these findings suggest that timing is impaired in PD, and that dopaminergic medication influences beat-based and non-beat-based timing differently. Judging the beat in music does not appear to be affected by PD or by dopaminergic medication.

## Introduction

Parkinson’s disease (PD) causes progressive motor and cognitive deficits, including deficits in timing (1, 2). Timing deficits in PD are likely related to dopaminergic dysfunction in the basal ganglia as the striatal dopaminergic system is known to be involved in timing (3, 4). One particular type of timing, beat-based timing, involves the timing of events relative to a regular interval, or a “beat,” such as in musical rhythm. In music, the beat is the regular, perceived emphasis to which listeners tend to synchronize their movements (e.g., by clapping their hands or tapping their feet). Beat-based timing activates the basal ganglia, among other cortical and subcortical regions of the motor system (5–9). There is mixed evidence regarding whether the basal ganglia have a *specific* role in beat-based timing compared to other types of timing. One study found that both beat-based (relative) and non-beat-based (absolute) timing was worse in participants from three clinical populations (not including PD) with impaired basal ganglia function, compared to control participants (10). This result was interpreted as supporting the “unified model” of timing (11), in which the basal ganglia play a central role in all types of timing, and not specifically in beat-based timing. However, another study found that patients with PD had a selective deficit in beat-based timing (12). Although both patients and controls discriminated metric simple (beat-based) rhythms more accurately than metric complex (non-beat-based) rhythms, patients with PD were significantly less accurate than controls for metric simple rhythms, but not for metric complex rhythms. This finding suggested a selective role for the basal ganglia in beat-based timing. However, all patients in the study were tested while ON dopaminergic medication, thus the influence of dopamine and dopaminergic medication on beat-based timing is not well understood.

For timing in PD generally, there is mixed evidence for dopaminergic medication’s influence. Dopaminergic medication improves timing production of participants with PD in a task using intervals in the range of 30–120 s, but not in the range of 250–2000 ms (13). In addition, participants with PD perform more similarly to control participants (with less timing variability) on a set of timing tasks while ON medication than while OFF (14). A study using behavioral and positron emission tomography (PET) found no effect of medication on PD patients’ ability to synchronize their tapping with an isochronous tones, although dopaminergic denervation was related to tapping accuracy (15). Another neuroimaging study (16) found that, although dopamine replacement therapy did not improve performance of patients with PD in a motor timing task, neural activity increased toward the level of controls in the dorsal putamen and supplementary motor area [regions associated with beat perception; (5)] during task performances. One study investigated the influence of dopaminergic medication on how well participants with PD detected beat structure in rhythms (17). Participants with PD and controls decided whether rhythms (that were either beat-based or non-beat-based) had a beat. Participants with PD did not significantly differ from controls, for either type of rhythm, although numerically participants with PD were worse than control participants at the task, and showed less difference in performance between beat-based and non-beat-based rhythms (control participants were better at recognizing that beat-based rhythms indeed had a beat than they were at recognizing that non-beat-based rhythms did not have a beat). As both groups had a small sample size (*n* = 9) and there was high variability between subjects, a real group difference in beat-based timing could have been missed. The study did find a small effect of dopaminergic medication: responses were faster when ON vs. OFF medication, and those responses were more accurate (though not statistically significantly). The task required explicit detection of beat structure in rhythms, similar to the Beat Alignment Test (BAT) in the present study. This explicit nature differs from the rhythm discrimination task used previously (12) and in the current study, for which an *implicit* influence of beat-based timing is expected: metric simple (beat-based) rhythms should elicit better performance than metric complex (non-beat-based rhythms), but no explicit awareness of the beat is required or assessed. Thus, there is mixed evidence for the influence of dopaminergic medication on timing in PD and limited evidence for its influence on beat-based timing in particular.

The uncertainty regarding dopaminergic medication’s influence on timing is partly related to the uncertainty regarding the extent to which cognitive deficits in PD (including timing) are associated with dopamine and would thus be modulated by dopaminergic medication. Besides deficient dopamine, other factors also contribute to cognitive deficits in the disease, including structural changes to the brain (18, 19), and accumulation of amyloid plaques and tau protein (20, 21). Moreover, the role of dopamine, and influence of dopaminergic medication, in cognition is variable, as previous studies show both improvement and worsening of different cognitive functions by medication, depending on task demands and individual differences in baseline dopamine levels (22, 23), as well as side of motor symptom onset (24).

The current study investigated the role of dopaminergic medication on beat-based timing in individuals with PD. We tested participants with PD on two beat perception tasks in two sessions: OFF and ON medication. We also tested control participants in two sessions, but did not give them medication, to assess practice effects. The two tasks were a rhythm discrimination task and the BAT [from the Goldsmiths Musical Sophistication Index; (25)]. In the discrimination task, participants decided whether two rhythms were the same or different. In several studies, the discrimination task has elicited better performance for metric simple rhythms (beat-based), compared to metric complex rhythms (non-beat-based) (5, 12, 26–28). This “beat-based advantage” is thought to depend on the beat-based timing (or relative timing) mechanism, which is thought to, in turn, depend on basal ganglia function (12). The second task, the BAT, presents excerpts of real music clips with a sequence of regular tones added to the music. The tones are either aligned or misaligned with the beat of the music, and participants decide whether the tones were on or off the beat of the music.

Both tasks assess beat perception; however, beat perception in the discrimination task arises solely on temporal information, without the rich variety of acoustic cues present in real music. The discrimination task also requires a comparison of two separately presented rhythms, introducing a working memory component. We hypothesized that if beat-based timing depends on basal ganglia function, and is thus impaired in PD, dopaminergic medication should improve discrimination of metric simple rhythms (but not metric complex rhythms). In contrast to the discrimination task, the BAT assesses beat perception in the context of real music, meaning that there are numerous musical features, unrelated to timing, that emphasize the beat (e.g., bass timbres or certain chord changes are more likely to occur on the beat). Beat perception in the BAT therefore does not rely solely on timing cues. The BAT has, to the best of our knowledge, not been used in the context of PD. If participants with PD perform worse than controls, it would provide converging evidence for a deficit in beat perception and suggest that other musical cues to the beat do not sufficiently compensate for that timing deficit. Similarly, if dopaminergic medication improves BAT performance in participants with PD, then beat perception in real musical contexts is likely dependent on basal ganglia function. Alternatively, if the groups do not differ, and/or there is no effect of medication on BAT performance, then beat perception arising from non-temporal cues likely does not rely on intact dopaminergic function of the basal ganglia.

## Materials and Methods

### Participants

Participants with PD were recruited from the St. Louis, MO, USA region to participate in an ongoing study investigating interventions for improving gait in PD. The data from these participants (*n* = 72, 30 female, mean age 66.8 years, SD = 9.4, mean of 2.18 years of musical training) are the baseline data, collected before any intervention, and only from participants who were taking dopaminergic medication at the time of testing. Controls (*n* = 70, 50 female, mean age 67.6 years, SD = 9.0, mean of 3.37 years of musical training) were recruited from the London, ON, Canada region. All participants scored at least 27 on the Mini Mental State Examination. Group differences in age and mean years of musical training were not statistically significant (*p* > 0.05). Participants’ highest achieved education levels were scored from 1 (high school) to 4 (advanced degree), and the groups did not differ on this measure (mean PD education = 2.79, SD = 1.05, mean control education = 2.90, SD = 1.07, *p* > 0.05). All participants provided informed, written consent in accord with procedures approved by the respective ethics boards at Western University and Washington University School of Medicine.

### Stimuli

**Figure 1:**
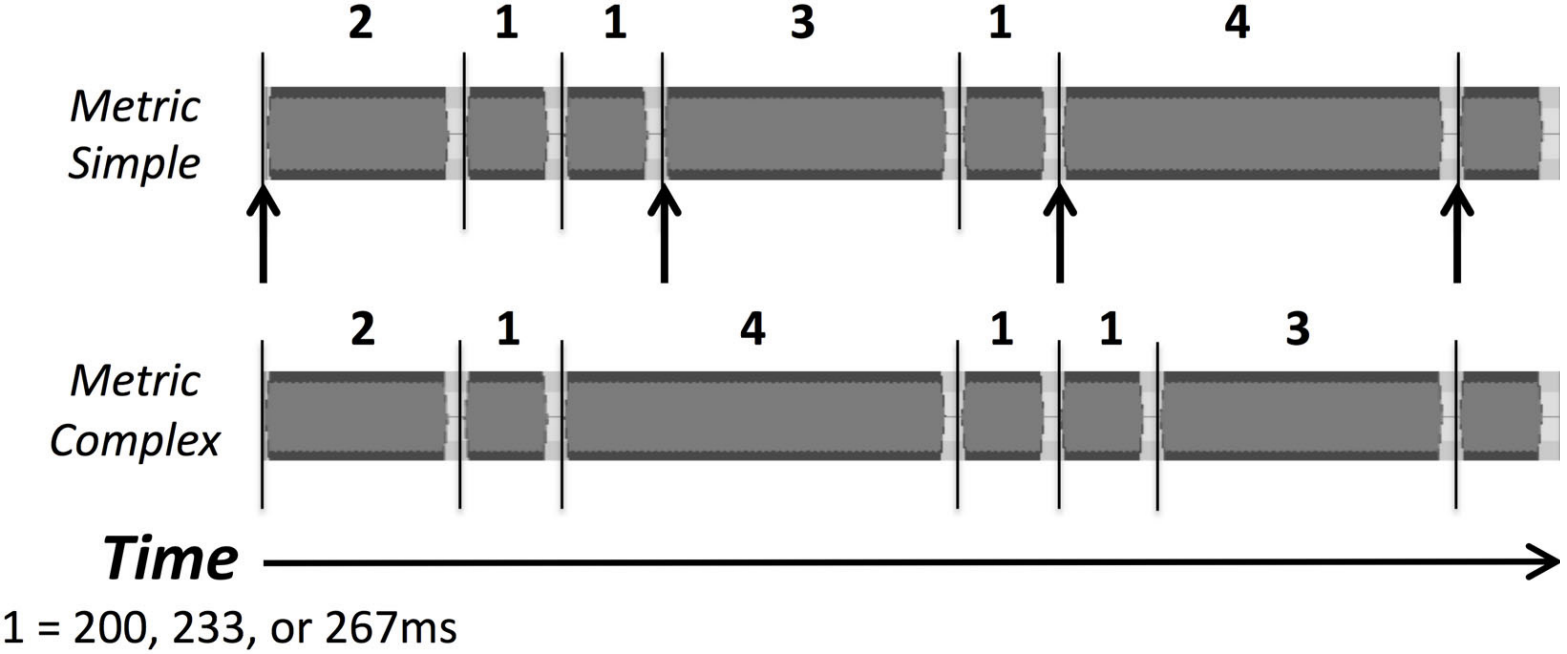
**Waveforms of examples of the two types of rhythmic sequences, metric simple and metric complex, used as stimuli in the rhythm discrimination task**. Numbers indicate the relative duration of intervals (1 = 200, 233, or 267 ms). Lines indicate tone onsets, and arrows indicate beat positions in the metric simple condition.

For the rhythm discrimination task, two types of rhythms were used: metric simple and metric complex (see Figure 1). The stimuli are described in detail elsewhere (12). Both types are composed of intervals that are related by small integer-ratios, and were presented in one of three tempi, corresponding to the shortest interval duration (i.e., rhythms consisted of interval durations of 1, 2, 3, and 4, in which “1” was equal to 200, 233, or 267 ms, “2” was twice the duration of “1,” etc.). The trials used were a subset of the trials used in a previous study (12), and are listed in Table 1.

**Table 1:** **Trials for rhythm discrimination task**.

Metric simple		Metric complex	
First rhythm	Second rhythm	First rhythm	Second rhythm
31413		33141	
41331	43131	41232	
112314		122142	
112422		124113	
211224	112224	132321	312321
222114		214311	124311
223113		323211	323121
311322		412212	412221
1122114	1121124	1132212	
1123113	1123131	1314111	1311411
2113113		2123211	1223211
2211114	2112114	2141211	
3121113	3121131	2331111	
3122112	1322112	3114111	1314111
4221111		3221112	3212112

Empty cells indicate that the second rhythm was the same as the first.

*Numbers indicate relative duration of intervals (1 = 200, 233, or 267 ms)*.

For the BAT [from the Goldsmiths Musical Sophistication Index; (25)], stimuli were 17 excerpts of music in which an isochronous tone sequence was embedded. The tone sequence could either be aligned with the beat of the music (“on beat,” four trials), faster or slower than the musical beat (period-shifted, eight trials), or at the same beat rate, but misaligned to the musical beat (phase-shifted, five trials).

### Procedure

Testing consisted of completing each task twice in the same day, with between 30 and 90 min separating the two testing sessions. Participants with PD were OFF medication in the first session (participants were asked to withhold all anti-Parkinson’s medication for at least 12 h prior to the session) and ON medication in the second session [all participants with PD were regularly taking levodopa (l-DOPA), typically in combination with carbidopa, except one participant who was taking rasagiline and pramipexole, one who was taking amantadine and pramipexole, and another who was taking ropinirole]. Between testing sessions, participants completed the MMSE and a questionnaire about their musical training. Additionally, participants with PD completed the Movement Disorders Society Unified Parkinson’s Disease Rating Scale [MDS-UPDRS; Ref. (29)].

Participants completed both tasks on laptops. Auditory stimuli were presented via headphones, and instruction text was presented on the laptop display. For the rhythm discrimination task, participants heard three consecutive rhythms (see Stimuli) and responded whether they thought the final rhythm was the same as the first two (which were always identical). During the two presentations of the first rhythm, the text “Original rhythm: First listen” and “Original rhythm: Second listen” were displayed, respectively, in white text. During presentation of the final, comparison, rhythm, the text “SECOND rhythm” was displayed in red text. Following presentation of the second rhythm, “Was the SECOND rhythm the same or different? If same, press (S). If different, press (D)” was displayed in white text. Participants then indicated whether they thought the second rhythm was the same as or different from the original rhythm. Four practice trials were completed before testing.

For the BAT, participants completed 17 trials (see Stimuli) in random order. Participants were given verbal instructions to listen to each music excerpt and to respond whether the embedded tone sequence was “ON” or “OFF” the beat of the music. During listening, the laptop display read “Please Listen,” and following each excerpt, it read “Are the tones on or off the beat? Press “y” for YES or “n” for NO on the keyboard.” Three practice trials were completed before testing.

### Analyses

Rhythm discrimination scores (proportion of correct trials) were initially analyzed in a 2 × 2 × 2 mixed analysis of covariance (ANCOVA) with the between-subjects factor of group (PD vs. control), and the within-subject factors of session (first vs. second session, also corresponding to OFF vs. ON medication for the participants with PD) and metricality of rhythms (metric simple vs. metric complex), and including the covariates musical training (years) and education level (both mean-centered separately for the two groups). Analyses were repeated without covariates that were non-significant and/or did not interact with other factors in the initial analysis.

As our primary research interest was the relationship between beat perception and dopaminergic medication [known to influence cognition in PD; (30)], and because we were unable to test the healthy controls ON medication, we conducted a separate 2 × 2 ANCOVA on the PD patient data alone. This ANCOVA included the within-subject factors medication (OFF vs. ON) and metricality (metric simple vs. metric complex), and the covariate of MDS-UPDRS (subscale III, off medication, mean-centered).

Furthermore, as only a subset of trials from the previous 2009 study (12) were used in the discrimination task (due to limitations of testing time), data from the 2009 study were reanalyzed to include only the subset of trials that were used in this current study. Results from the 2009 study and current study (ON and OFF medication, separately) were compared using independent samples *t* tests.

Beat alignment test scores (proportion of correct trials) were analyzed in a 2 × 2 mixed ANCOVA with the between-subjects factor of group (control vs. PD) and within-subject factor of session (first vs. second session, also corresponding to OFF vs. ON medication for the participants with PD), and musical training and education (both mean-centered, separately for the two groups) as covariates.

## Results

### Rhythm Discrimination

**Figure 2:**
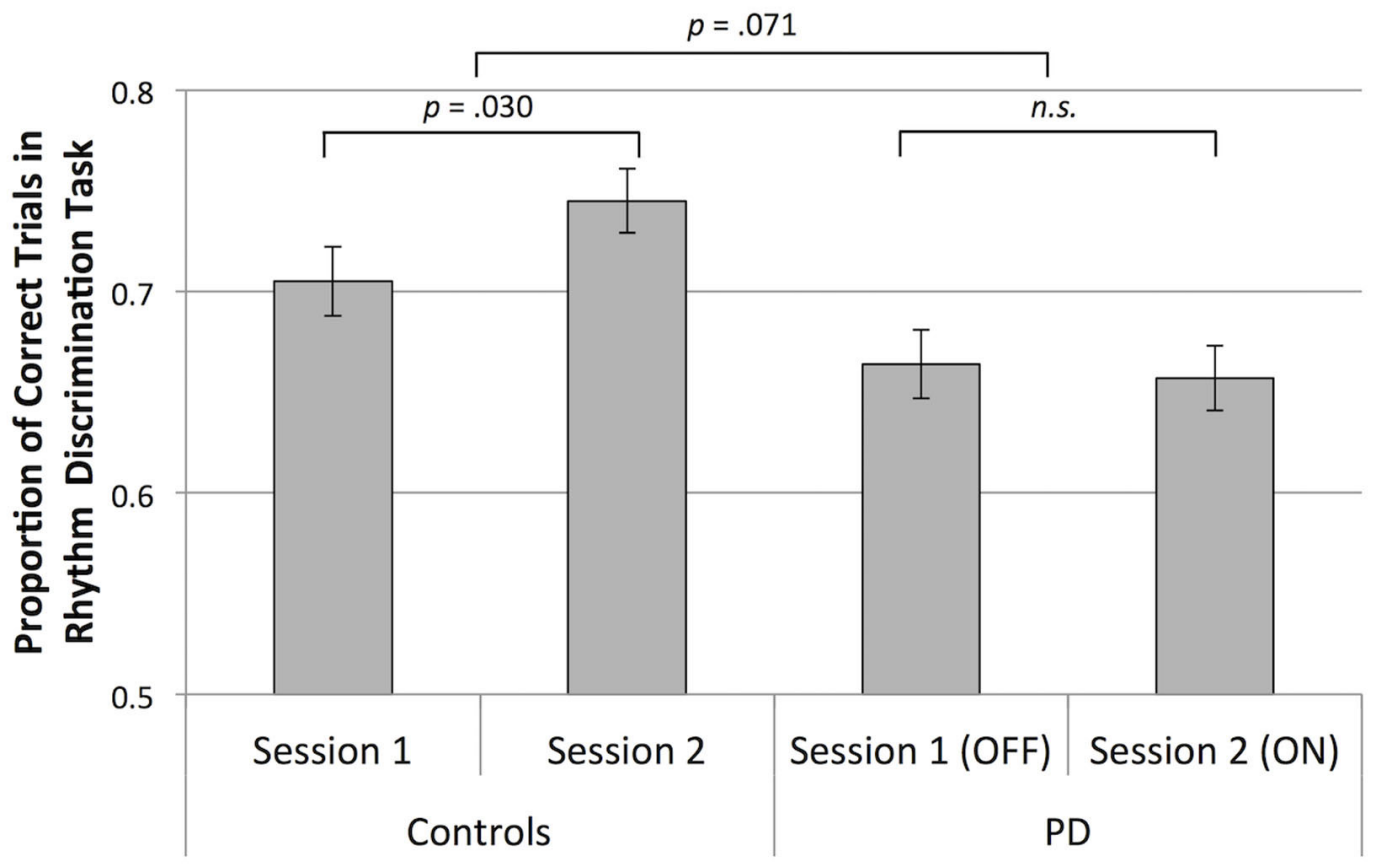
**Mean rhythm discrimination scores (adjusted for musical training and education) collapsed across rhythm type for both groups, in both sessions**. Error bars indicate ± 1 SEM.

**Figure 3:**
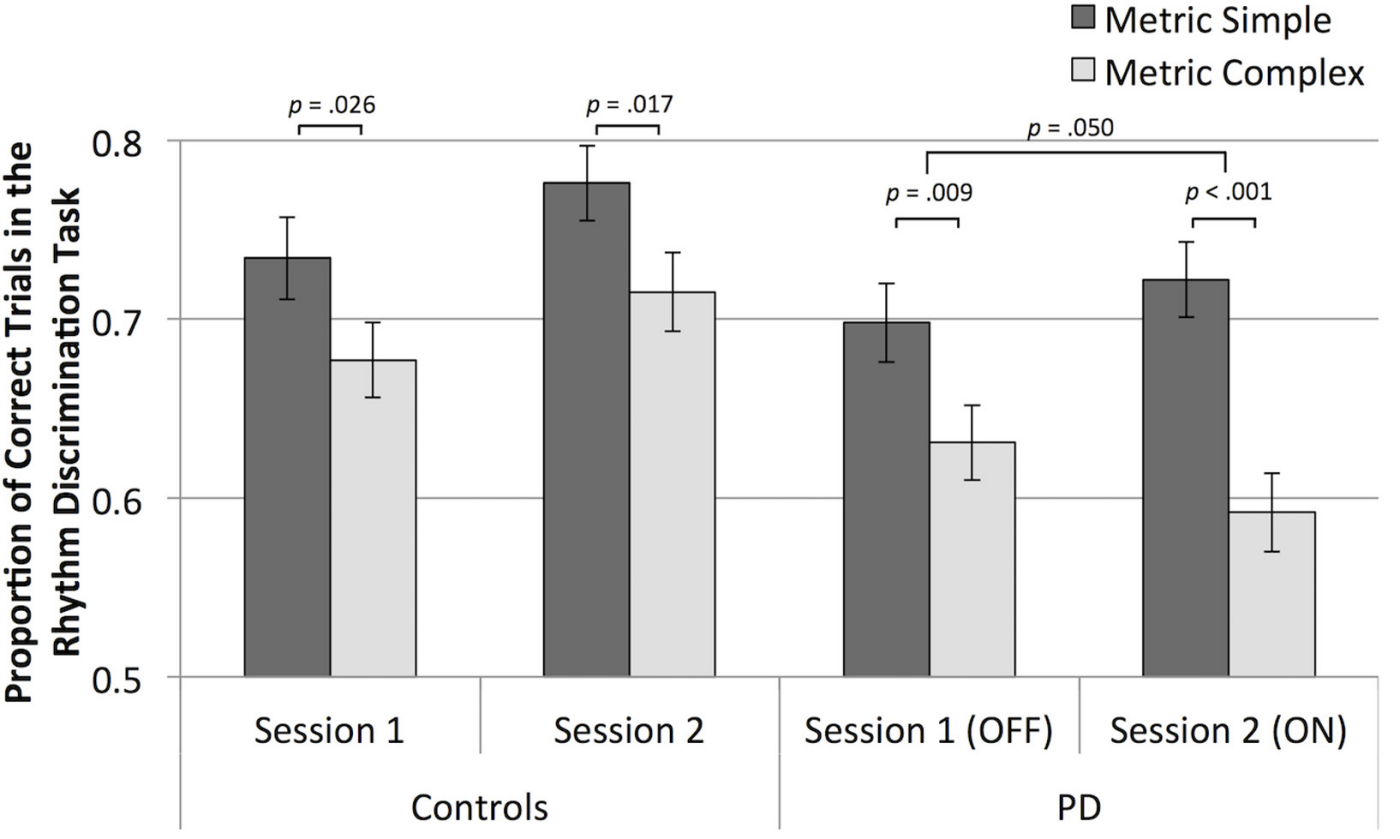
**Mean rhythm discrimination scores (adjusted for musical training and education) for both groups, in both sessions, and for both metrical types of rhythms**.

Participants with PD were worse than control participants at discriminating rhythms [main effect of group, *F*(1,136) = 10.86, *p* = 0.001, $\eta_{p}^{2}=0.0\text{74}$]. In the second session, control participants performed better than in the first session, and participants with PD did not show this improvement [a statistical trend toward an interaction of group and session, *F*(1,136) = 3.31, *p* = 0.071, $\eta_{p}^{2}=0.0\text{24}$], confirmed by follow-up paired *t* tests comparing average scores within each session for healthy controls, [*t*(68) = 2.22, *p* = 0.030, and for participants with PD, *p* > 0.05], as shown in Figure 2. Overall, discrimination was better for metric simple rhythms than for metric complex rhythms [main effect of metricality, *F*(1,136) = 36.50, *p* < 0.001, $\eta_{p}^{2}=0.\text{212}$]. This effect of metricality was present for both groups in both sessions (*p* < 0.05, in all cases), as shown in Figure 3. Regarding musical training, there was a statistical trend toward those more training being better at discriminating rhythms [*F*(1,136) = 3.77, *p* = 0.054, $\eta_{p}^{2}=0.0\text{27}$]. Although education level was not a significant covariate, there was a trend toward education interacting with metricality [*F*(1,136) = 3.63, *p* = 0.059, $\eta_{p}^{2}=0.0\text{26}$]. Follow-up comparisons of these data show that trends were in opposite directions: those with more education tended to do better with metric complex rhythms and slightly worse with metric simple rhythms. The three-way interaction between group, session, and metricality did not reach significance (*p* > 0.1).

Analysis of only the data from participants with PD shows that metric simple rhythms were discriminated better than metric complex rhythms [main effect of metricality, *F*(1,69) = 29.81, *p* < 0.001, $\eta_{p}^{2}=0.\text{3}0\text{2}$], as is evident in Figure 3. The effect of metricality was larger when participants were ON medication compared to OFF medication [interaction of metricality and medication, one-tailed, as the direction of differences was hypothesized, *F*(1,69) = 2.77, *p* = 0.050, $\eta_{p}^{2}=0.0\text{39}$]. A paired *t*-test of the difference scores (metric simple minus metric complex, adjusted for UPDRS) confirmed a significantly greater difference while ON vs. OFF medication [*t*(70) = 1.69, *p* = 0.048]. Follow-up *t* tests indicated that metric simple scores numerically increased and metric complex scores numerically decreased from OFF to ON sessions, although neither change was statistically significant (*p* > 0.05).

**Figure 4:**
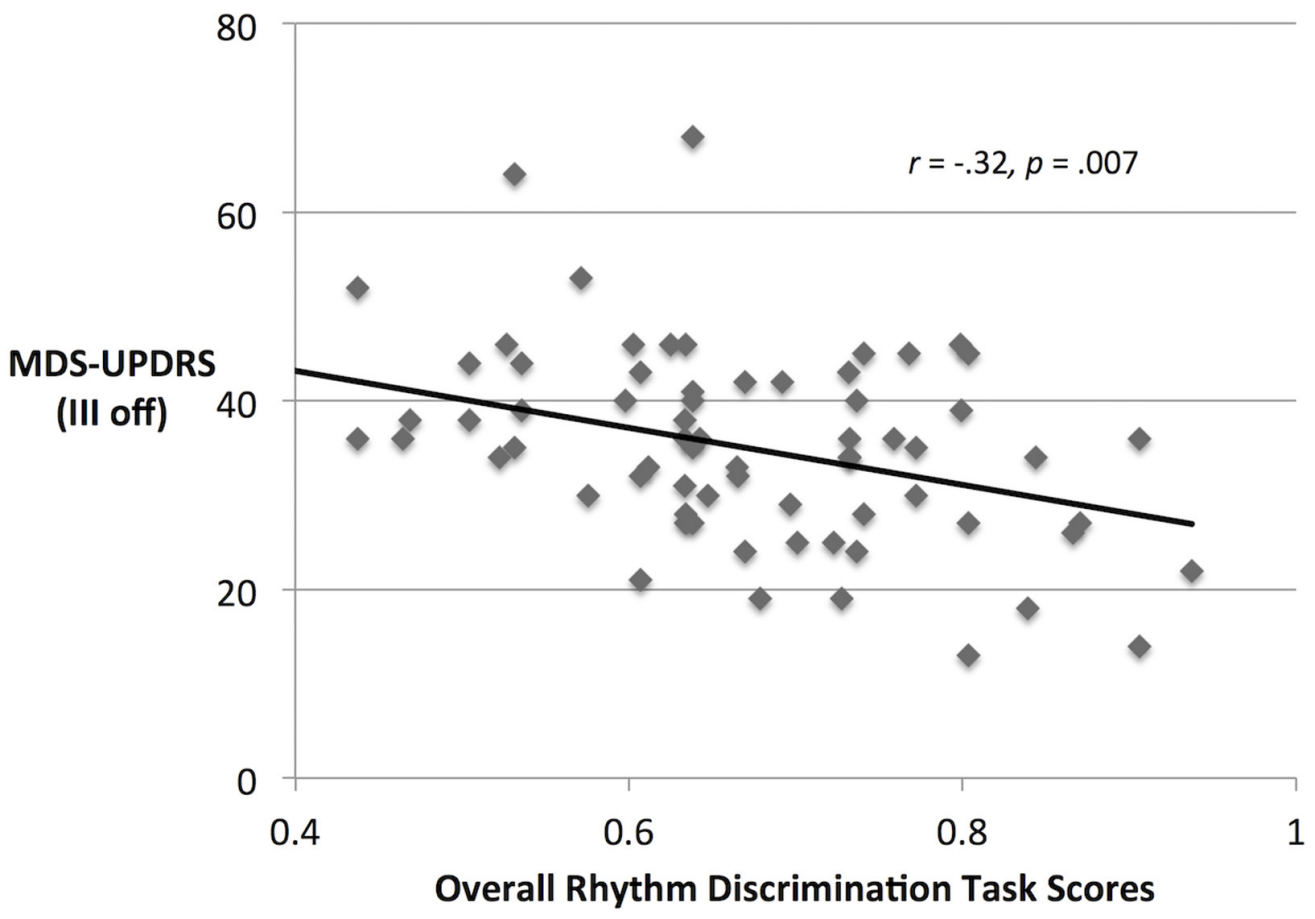
**MDS-UPDRS scores and rhythm discrimination scores from participants with PD (discrimination scores are collapsed over OFF and ON medication conditions)**.

Movement Disorders Society Unified Parkinson’s Disease Rating Scale scores (off medication) significantly covaried with overall discrimination performance [*F*(1,69) = 11.49, *p* = 0.001, $\eta_{p}^{2}=0.\text{143}$], as shown in Figure 4. MDS-UPDRS scores and mean discrimination scores (averaged over sessions and metricalities) negatively correlate (*r_*Sp*_* = −0.32, *p* = 0.007). Although there was no significant interaction between MDS-UPDRS, medication, and task performance, exploratory analysis showed that performance negatively correlated with MDS-UPDRS scores both OFF and ON medication (*r_*Sp*_* = −0.28, *p* = 0.020 and *r_*Sp*_* = −0.36, *p* = 0.002, respectively).

**Figure 5:**
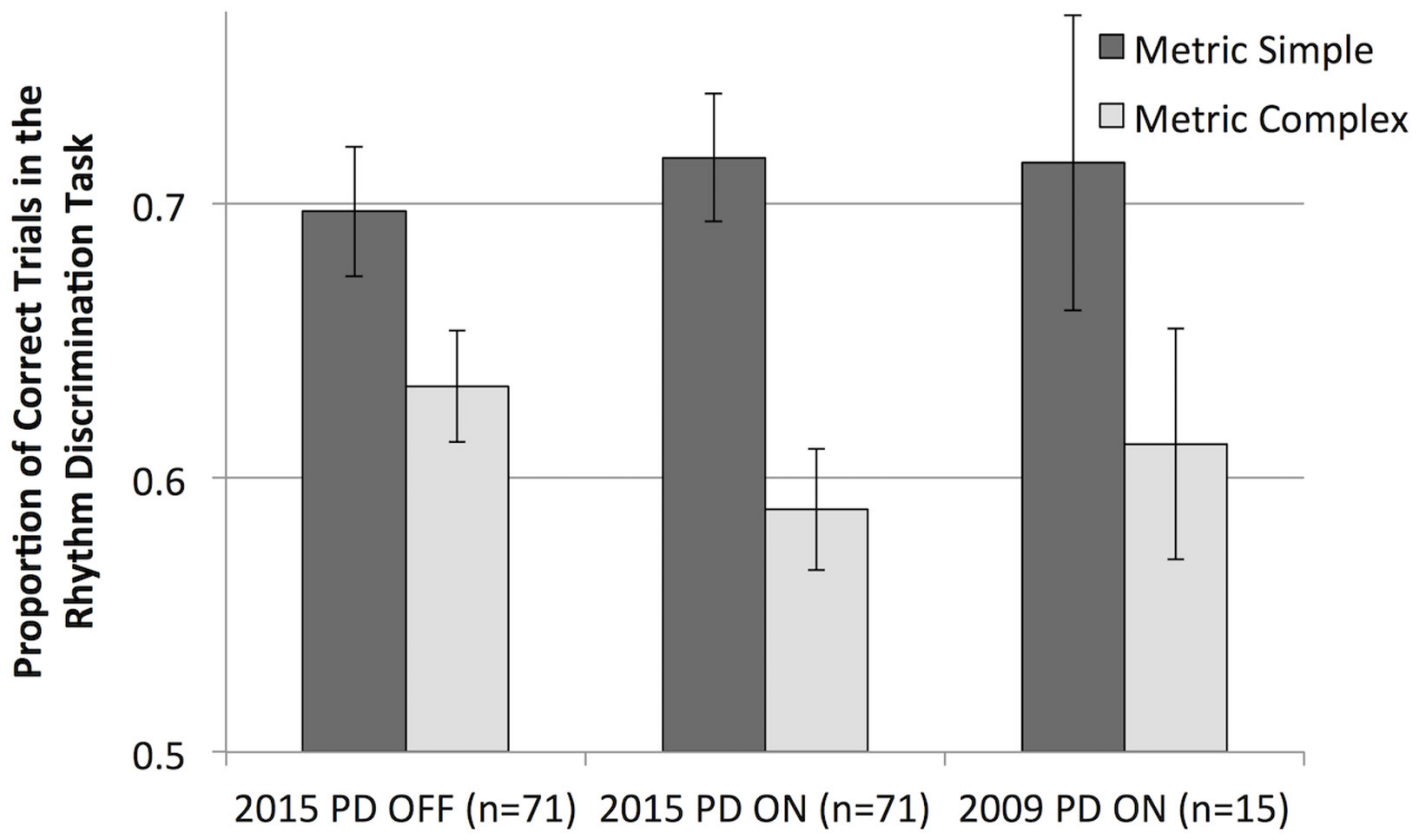
**Mean rhythm discrimination scores (unadjusted) from the current sample of participants with PD (OFF and ON dopaminergic medication) and from a previous study (ON medication only, and recalculated to include only the trials used in the current study)**.

We compared the results from our current participants with PD to the results of the 2009 study using the same task (with previous data recalculated to include only the same trials used in the present study). The 2009 sample was participants with PD, ON medication with one session of testing [*n* = 15; (12)]. The current ON medication sample was numerically more similar to the 2009 sample (also ON medication) than to the current OFF medication sample. Overall, however, independent samples *t* tests comparing the 2009 sample and the current sample ON and OFF medication (separately) show no statistically significant differences (*p* > 0.05) between discrimination scores for either metric simple or metric complex rhythms (see Figure 5). Thus, when including identical trials between the 2009 sample and the current sample, the two groups of patients did not differ in performance.

### Beat Alignment Test

**Figure 6:**
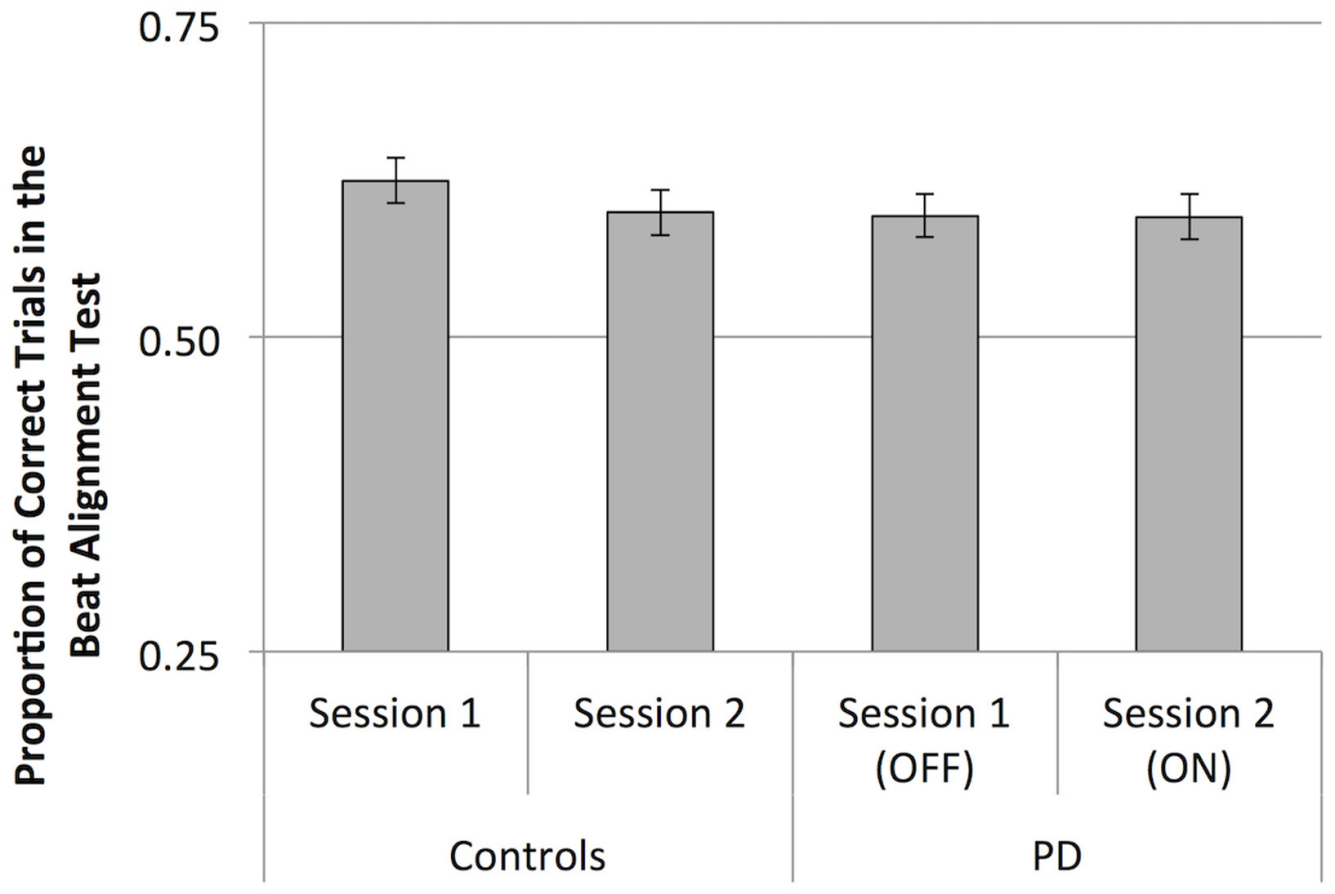
**Mean scores (proportion of correct trials) for the Beat Alignment Test**. Error bars indicate ± 1 SEM.

Performance on the BAT did not differ between groups, or between sessions (see Figure 6). BAT scores were not associated with musical training or education, or MDS-UPDRS scores in participants with PD. Furthermore, BAT scores did not correlate with mean discrimination scores (*p* > 0.05). When analyzing data from participants with PD alone, we found no effect of medication or interactions involving medication (*p* > 0.05).

## Discussion

Overall, the rhythm discrimination task was sensitive to timing deficits in PD: participants with PD performed worse than control participants, and participants more severely affected by PD (those with higher MDS-UPDRS scores) did worse than those less affected. Furthermore, control participants improved over the two sessions while participants with PD did not, suggesting that in addition to a deficit in timing, they may be less able to learn from repetition of the timing task. Although we did not expect this result, and do not consider it a primary finding of the study, it is consistent with previous evidence of learning deficits in PD. For example, patients with PD show less consolidation than controls after learning a motor control task (31), and slower re-learning after disruption of a previously learned motor task (32). Moreover, dopaminergic medication can change the nature of learning in PD (33–35), possibly contributing to the lack of improvement in discrimination task performance from session 1 (OFF medication) to session 2 (ON medication) in PD. However, as the repetition of the task is confounded with medication status in participants with PD (the first session was always OFF medication, followed by ON medication in the second session), we cannot fully disentangle effects of repetition and medication in participants with PD.

The BAT was not sensitive to timing deficits in PD, as performance on the task by control participants and those with PD did not significantly differ. The additional information in real music may give listeners with PD sufficient cues regarding the beat, such that beat-based timing deficits do not impair task performance. In addition to the other musical cues to the beat that are present in the BAT, participants compare simultaneously presented sequences (the beat in the musical stimulus, and the overlaid tone sequence). The discrimination task, by comparison, requires a memory-based judgment, which requires the encoding, rehearsal, and retrieval of a rhythm. These processes involve the internal generation of rhythms, which is supported by the presence of temporal structure, such as the beat. Thus, the difference in cognitive processes required by the BAT and the rhythm discrimination task may underlie the difference in findings between tasks regarding the particular nature of timing deficits in PD. Consistent with this interpretation, performance on the BAT and discrimination task did not correlate, suggesting that these tasks are indeed sensitive to different aspects of beat perception, and rely on different underlying cognitive processes. Although BAT performance was numerically better for control participants than participants with PD, the large sample sizes suggest that any potential difference between patients and controls is small.

Regarding the discrimination task, we found mixed results. Contrary to our expectation, we did not see a clear deficit in beat perception associated with PD. Participants with PD performed better on the task for metric simple (beat-based) rhythms than for metric complex (non-beat-based) rhythms, similar to control participants. This is at odds with the finding from a previous study that used the same two types of rhythms in the same rhythm discrimination task, showing that the beat-based advantage (superior discrimination of metric simple rhythms) was significantly reduced in PD compared to controls. However, when results from the previous study were recalculated to include only the exact same trials used in the current study, the difference in performance between metric simple and metric complex rhythms increased, and closely resembled that of the current data (see Figure 5), suggesting that our current study did indeed replicate the previous results. Moreover, the previous study’s recalculated data most closely resembles the ON medication data from the current study. This is notable as participants in the previous study were ON medication. The dependence of performance on the specific trials included demonstrates a potential limitation of discrimination tasks: performance is dependent not just on condition differences (rhythms are easier to encode and maintain when participants perceive a beat, therefore performance is generally better for beat-based rhythms) but also the specific nature of the discrimination being made. The task required participants to detect whether a change occurred in the rhythm, and the change, when present, was always a transposition (or swapping) of two time intervals. For example, the rhythm 211314 could become 211134. Some transpositions are easier to detect than others. Changes to the beginning or end of a rhythm are easier to detect than those in the middle because of primacy and recency effects (36). In addition, transposition of disparate intervals (e.g., 3 and 1) may be easier to detect than transposition of more similar intervals (e.g., 3 and 4). Thus, by reducing the number of trials selected for the current study, the results could be more influenced by these trial-specific differences that are not related to beat perception, but are instead related to the specific nature and location of the change in the rhythm. However, as the current results do not differ from the previous results (which used a much larger set of trials) when reanalyzed to include only the same trials, we feel it reasonable to interpret the current findings as replicating the previous finding that beat-based timing is impaired in PD, although trial selection influenced the exact pattern of results. Further support for a beat-based timing impairment needs to be acquired. This may be best accomplished by using different tasks, such as rhythm reproduction that do not have the limitations present in discrimination tasks.

For participants with PD, the difference in discrimination performance between metric simple and metric complex rhythms increased when ON vs. OFF dopaminergic medication. The data therefore suggest that medication influences beat-based timing in PD, although the pattern of the influence is complex. In particular, the worsening of performance for metric complex (non-beat-based) rhythms was unexpected. The improvement of performance for beat-based rhythms may be due to dopaminergic medication improving basal ganglia function, as they are thought to play a critical role in beat perception. Another possibility is that dopaminergic medication biases participants to search for a beat structure. This bias would improve performance for beat-based rhythms, in which a beat structure can be detected, and therefore searching for it is beneficial, but the same bias would worsen performance for metric complex rhythms, in which the beat structure is difficult to find, and attempting to search for it distracts from using another, better, strategy to remember the rhythms. As mentioned above, task repetition (first vs. second session) is confounded with medication (OFF vs. ON); however, the metricality-dependent change in performance (improved performance for metric simple rhythms and worsened performance for metric complex rhythms) is less likely due to repetition than to medication. An expected effect of repetition would be improved performance for one or both types of rhythms, but not worse performance. As such, we interpret the overall lack of improvement at the task as a deficit in learning the task (compared to control participants’ overall improvement from the first to second session), but the different direction of performance change for metric simple and metric complex rhythms as an effect of medication on beat-based timing.

Overall, these data present further evidence that timing is impaired in individuals with PD (and worsens with severity of the disease) that beat-based timing may be particularly impaired in PD, as we replicated findings from Grahn and Brett (12), which used a more complete set of trials (less subject to trial-specific effects) to show a lack of beat-based advantage, and that, consistent with previous work (17), dopaminergic medication may improve beat-based timing in PD.

## Author Contributions

All authors contributed to design. DC and KP collected data. DC analyzed data, and all authors contributed to writing.

## Conflict of Interest Statement

The authors declare that the research was conducted in the absence of any commercial or financial relationships that could be construed as a potential conflict of interest.
